# Data Augmentation for Deep Neural Networks Model in EEG Classification Task: A Review

**DOI:** 10.3389/fnhum.2021.765525

**Published:** 2021-12-17

**Authors:** Chao He, Jialu Liu, Yuesheng Zhu, Wencai Du

**Affiliations:** ^1^Shenzhen EEGSmart Technology Co., Ltd., Shenzhen, China; ^2^School of Electronic and Computer Engineering, Peking University, Beijing, China; ^3^Institute for Data Engineering and Sciences, University of Saint Joseph, Macao, Macao SAR, China

**Keywords:** brain-computer interface, EEG, deep neural networks, data augmentation, classification

## Abstract

Classification of electroencephalogram (EEG) is a key approach to measure the rhythmic oscillations of neural activity, which is one of the core technologies of brain-computer interface systems (BCIs). However, extraction of the features from non-linear and non-stationary EEG signals is still a challenging task in current algorithms. With the development of artificial intelligence, various advanced algorithms have been proposed for signal classification in recent years. Among them, deep neural networks (DNNs) have become the most attractive type of method due to their end-to-end structure and powerful ability of automatic feature extraction. However, it is difficult to collect large-scale datasets in practical applications of BCIs, which may lead to overfitting or weak generalizability of the classifier. To address these issues, a promising technique has been proposed to improve the performance of the decoding model based on data augmentation (DA). In this article, we investigate recent studies and development of various DA strategies for EEG classification based on DNNs. The review consists of three parts: what kind of paradigms of EEG-based on BCIs are used, what types of DA methods are adopted to improve the DNN models, and what kind of accuracy can be obtained. Our survey summarizes the current practices and performance outcomes that aim to promote or guide the deployment of DA to EEG classification in future research and development.

## Introduction

As a key tool to capture the intention of brain activity, electroencephalography (EEG) can be used to measure rhythmic oscillations of the brain and reflect the synchronized activity of substantial populations of neurons (Atagün, 2016). The rhythmic oscillation is closely related to the state change of the nerve center that directly reflects the mental activity of the brain (Pfurtscheller, 2000; Villena-González et al., 2018). The brain-computer interface (BCI) is one of the typical applications used as a communication protocol between users and computers that does not rely on the normal neural pathways of the brain and muscles (Nicolas-Alonso and Gomez-Gil, 2012). Based on the generation types of EEG, BCIs can be divided into three types: non-invasive BCIs, invasive BCIs, and partially invasive (Rao, 2013; Levitskaya and Lebedev, 2016). Due to the low risk, low cost, and convenience, the EEG-based non-invasive BCIs are the most popular type of BCIs and are the main type discussed in this article.

During the execution of the interaction, the automatic classification of EEG is an important step toward making the use of BCI more practical in applications (Lotte et al., 2007). However, some limitations present challenges for classification algorithms (Boernama et al., 2021). Firstly, EEG signals have weak amplitudes and are always accompanied by irrelated components which suffer from a low signal-to-noise ratio. Secondly, the essence of EEG is the potential change of cluster activity of neurons which is a non-stationary signal. The technologies of machine learning and non-linear theory are widely used for EEG classification in current research (Lotte et al., 2018). However, a long calibration-time and weak generalization ability limits their application in practice.

In the past few years, deep neural networks (DNNs) have achieved excellent results in the field of image, speech, and natural text processing (Hinton et al., 2012; Bengio et al., 2013). The features can be automatically extracted from the input data by successive non-linear transformations based on hierarchical representations and mapping. Due to their ability to minimize the interference of redundant information and non-linear feature extraction, EEG decoding based on DNNs has attracted more and more attention. However, one of the prior conditions to obtain expected results is the support of large-scale datasets that could ensure the robustness and generalization ability of DNNs (Nguyen et al., 2015). There are still some challenges for EEG collection. First, it is difficult to collect large-scale data due to strict requirements for the experimental environment and subjects that may cause overfitting and increase the structural risk of the model (Zhang D. et al., 2019). More than that, EEG signals are highly susceptible to change in psychological and physiological conditions that cause high variability of feature distribution across subject/sessions (Zhang D. et al., 2018). It not only reduces the accuracy of the decoding model, but also limits the generalization of the model in the independent test set.

**FIGURE 1:**
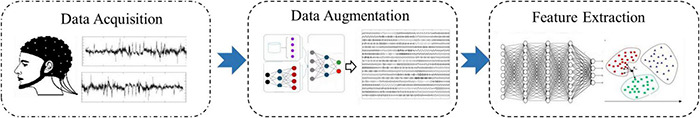
The framework of EEG classification using DA strategy. Different color represents different classs.

One promising approach is regularization (Yu et al., 2008; Xie et al., 2015), which could effectively improve the generalization ability and robustness for DNNs. There are three ways to achieve regularization, including adding term into loss function (e.g., L2 regularization), directly in the model (e.g., dropout, batch normalization, kernel max norm constraint), and data augmentation (DA). Compared to the first two approaches, DA solves the problem of overfitting by using a more comprehensive set of data to minimize the distance between the training and test dataset. This is especially useful for EEG signals where the limitation of small-scale datasets greatly affects the performance of classifiers. Therefore, researchers are increasingly concerned with optimization for deep learning (DL) models using DA in the task of EEG classification. The framework of the methodology is shown in Figure 1.

The rest of the article is organized as follows. The search methods for identifying relevant studies is described in detail in section “Method.” In section “Results,” the basic concept and specific methods of DA in EEG classification based on DNNs are presented. Section “Discussion” discusses the current research status and challenges. Finally, conclusions are drawn in section “Conclusion.”

## Method

A wide literature search from 2016 to 2021 was conducted through Web of Science, PubMed, and IEEE Xplore. The keywords used for the search contain DA, EEG, deep learning, DNNs. Table 1 lists the collection criteria for inclusion or exclusion.

**TABLE 1:** Collection criteria for inclusion or exclusion.

Inclusion criteria	Exclusion criteria
• Published within the last 5 years	• Research for invasive EEG, electrocorticography (ECoG), magnetoencephalography (MEG), source imaging, fMRI, and so on, or joint studies with EEG
• A focus on non-invasive EEG signals	• No specific description of DA
• A specific explanation of how to apply DA to EEG signal	
• At least one DNN is included for the classifier • EEG used in the BCI task or the detection of sleep states or epileptic seizures task	

**FIGURE 2:**
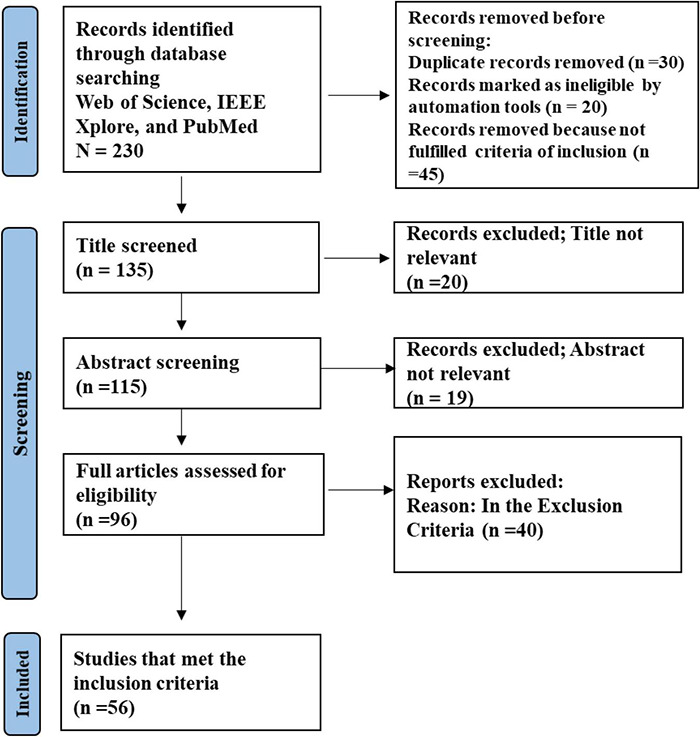
The search method for identifying relevant studies.

This review was conducted following PRISMA guidelines (Liberati et al., 2009). Results are summarized in a flowchart in Figure 2. The flowchart identifies and narrows down the collection of related studies. Duplicates between all datasets and studies that meet the exclusion criteria are excluded. Finally, 56 papers that meet the inclusion criteria are included.

## Results

### Concepts and Methods for Data Augmentation

Data augmentation aims to prevent the overfitting of the DNN model by artificially generating new data based on existing training data (Shorten and Khoshgoftaar, 2019). There are three main strategies of this technology: basic image manipulations, deep learning, and feature transformation. The first approach performs augmentation directly in the input space while the last two methods realize DA based on the feature space of datasets. Here, we briefly describe these methods in the following parts.

#### Data Augmentation Based on Image Manipulations

Data augmentation based on image manipulations perform simple transformations using geometric features in an intuitive and low-cost way. Typical methods could be divided into the following categories.

##### Geometry Transformations

The geometric features of images are generally a visual representation of the physical information that contains both direction and contour elements (Cui et al., 2015; Paschali et al., 2019). Common operations include:

##### Flipping

This method is realized by rotating the image along the horizontal or vertical axis under the premise that the size of the matrix was consistent.

##### Cropping

The operation of cropping can be realized by cropping the central patch of images randomly and then mixing the remaining parts.

##### Rotation

Data augmentation rotation is realized by rotating images along some coordinate axis. How to select rotation parameters is an important factor that affects the enhancement effect.

##### Photometric and Color Transformations

Performing augmentations in the color channels’ space is another method to implement practically (Heyne et al., 2009). During the operation, the raw data are converted to a form of the power spectrum, stress diagram, and so on. They represent the distribution of spatial features.

##### Color Transformations

Color transformation realizes the generation of new data by adjusting the RGB matrix.

##### Noise Injection

Another approach to increase the diversity of data is injecting random matrices into the raw data, which are usually derived from Gaussian distributions (Okafor et al., 2017).

#### Data Augmentation Based on Deep Learning

Augmentation methods by image manipulations perform the transformation in input space of data. However, these approaches cannot take advantage of underlying features of data to perform augmentation (Arslan et al., 2019). Recently, a novel DA method has attracted the attention of researchers. It applies DNNs to map data space from high-dimensional to low-dimensional and realize feature extraction to reconstruct the artificial data (Cui et al., 2014). There are two typical deep learning strategies for DA: autoencoder (AE) and generative adversarial networks (GAN).

##### Autoencoder and Its Improved Version

**FIGURE 3:**
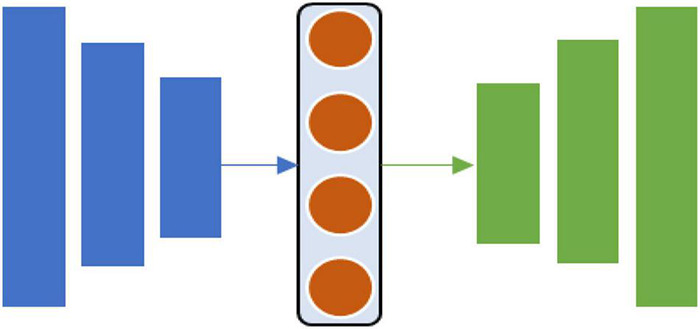
The structure of the autoencoder. Blue represents input layers and green represents output layers.

As shown in Figure 3, an AE is a feed-forward neural network used to encode the raw data into low-dimensional vector representations by one-half of the network and to reconstruct these vectors back into the artificial data using another half of the network (Yun et al., 2019).

**FIGURE 4:**
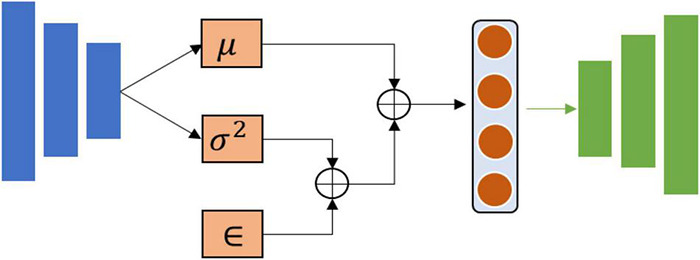
The structure of variational autoencoder. Blue represents input layers and green represents output layers.

To obtain the expected generated data, a variational autoencoder (VAE) is proposed to improve the performance of the autoencoder. Compared with AE, VAE ensure that generated data is subject to specific probability distribution by adding constraints into the structure (Figure 4).

Where μ is the mean value of probability distribution, σ^2^ represents variance, and ∈ is deviation.

##### Generative Adversarial Networks and Their Improved Version

**FIGURE 5:**
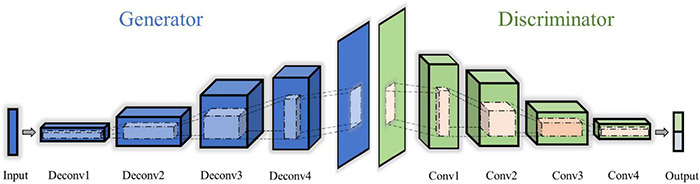
The structure of generative adversarial networks.


Generative adversarial networks refer to artificially generating data based on the principle of adversarial learning. As shown in Figure 5, it performs a competition between bilateral networks to achieve a dynamic balance that learns the statistical distribution of the target data (Deng et al., 2014). The optimization problem of GAN can be defined as follows:

(1) $${\mathrm{Min}}_{G}{\mathrm{Max}}_{D}V\left(D,G\right)=E_{x∼p\left(x\right)}\left[\mathrm{logD}\left(x;\theta_{G}\right)\right]+E_{z∼p\left(z\right)}\left[\log\left(1-D\left(x;\theta_{G}\right)\right)\right]$$

Where ***p(x)*** is the distribution of training data and **D**(**x**;θ_**G**_) is the discriminative model used to estimate the probability distribution ***p*** (•) between generated data ***z*** of real data ***x***. ***V*** represents the value function and ***E*** is the expected value. In the process of training stage, the goal of GAN is to find the Nash equilibrium of a non-convex game with high-dimensional parameters. However, the optimization process of the model does not constraint for loss function that is easy to generate a meaningless output during the training stage. To address the issue and expand its application scope, the researchers proposed improved structures such as deep convolution GAN, conditional GANs, cycle GANs, and so on (Goodfellow et al., 2014). Amongst these new architectures for DA, DCGAN employed the CNNs to build the generator and discriminator networks rather than multilayer perceptron that expands more on the internal complexity than GAN (Radford et al., 2015). To improve the stability of the training process, an additional cycle-consistency loss function was proposed to optimize the structure of GAN, which was defined as cycle GANs (Kaneko et al., 2019). Conditional GANs effectively alleviate the limitations with mode collapse by adding a conditional vector to both the generator and the discriminator (Regmi and Borji, 2018). Another architecture of interest is known as Wasserstein GAN (WGAN). This architecture employed Wasserstein distance to measure the distance between generated data with real data rather than Jensen–Shannon or Kullback–Leibler divergence to improve the training performance (Yang et al., 2018).

#### Data Augmentation Based on Feature Transformation

Compared with the method of image manipulations and deep learning, feature transformation performs DA using spatial transformation of features in low dimensions that generate artificial data with a diverse distribution. However, a few studies have reported related methods. A novel spatial filtering method has been proposed to generate data using a time-delay strategy by combining it with a common spectral-spatial pattern (CSSP; Blankertz and BCI Competition, 2005). Another study applied empirical mode decomposition to divide EEG into multiple modes for DA (Freer and Yang, 2019).

**FIGURE 6:**
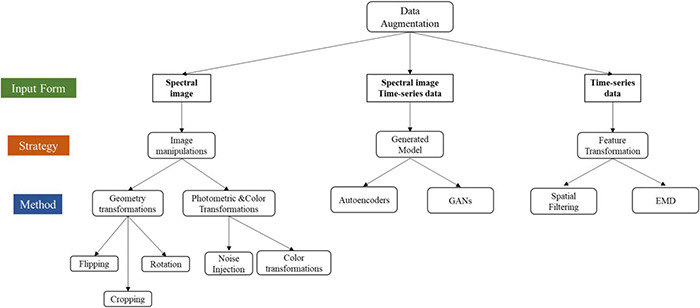
A taxonomy of data augmentation in EEG decoding.

To clearly show the taxonomy of DA, Figure 6 briefly integrated all the DA methodologies collected in this review.

### Typical EEG Paradigms

Based on the form of interaction, the BCIs can be divided into two types: active type and passive type. Among them, active BCI is defined as a neural activity to a specific external stimulus that contains three typical paradigms: Motor imagery (MI), visual evoked potentials (VEP), and event-related potentials (ERP). MI is a mental process that imitates motor intention without real output. Different imagery tasks can activate the corresponding region of the brain, while this activation can be reflected by various feature representations of EEG (Bonassi et al., 2017).

Visual evoked potentials are continuous responses from the visual region when humans receive flashing visual stimuli (Tobimatsu and Celesia, 2006). When external stimuli are presented in a fixed frequency form, the visual region is modulated to produce a continuous response related to this frequency, i.e., Steady-State Visually Evoked Potentials (SSVEP; Wu et al., 2008).

Event-related potentials refers to a potential response when receiving specific stimulus such as visual, audio, or tactile stimulus (Luck, 2005).

Compared with active BCI, passive BCI aims to output the EEG signals from subjects’ arbitrary brain activity, which is a form of BCI that does not rely on voluntary task (Roy et al., 2013; Arico et al., 2017).

In this section, we review recent reports for DA in EEG classification based on DNNs.

### Data Augmentation Strategy for EEG Classification

In recent years, scientific interest in the field of the application of DA for EEG classification has grown considerably. Abdelfattah et al. (2018) employed recurrent GANs (RGAN) to improve the performance of classification models in the MI-BCI tasks. Different from the structure of GAN, they applied recurrent neural networks to replace generator components. Due to its ability to capture the time dependencies of signals, RGAN show great advantages in time-series data generating. The classification accuracy was significantly improved after DA through the verification of three models.

Zhang et al. (2020) carried out the research of image augmentation using deep convolution GAN (DCGAN) that replace pooling layers with Fractional-Strided Convolutions in the generator and strided convolutions in the discriminator. Considered the rule of feature distribution, they transformed time-series signal to spectrogram form and applied adversarial training with convolution operation to generate data. Meanwhile, they discussed the performance of different DA models and then verified that the generated data by DCGAN show the best similarity and diversity.

Freer and Yang (2019) proposed a convolutional long-short term memory network (CLSTM) to execute binary classification for MI EEG. To enhance the robustness of the classifier, they applied noise injection, multiplication, flip, and frequency shift to augment data, respectively. Results show average classification accuracy could obtain 14.0% improvement after DA.

Zhang Z. et al. (2019) created a novel DA method in the MI-BCI task in which they applied empirical mode decomposition (EMD) to divide the raw EEG frame into multiple modes. The process of decomposition was defined as:


(2) $$x\left(t\right)=\sum_{n=1}^{n}{\mathrm{IMF}}_{n}\left(t\right)+r_{s}\left(t\right)$$

Where *x*(*t*) is recovered signal by EMD, *IMF* represents intrinsic mode functions, *s* represents the number of *IMFs*, and *r*_*s*_(*t*) is the final residual value. In the training stage, they mixed IMFs into the intrinsic mode functions to generate new data and then transformed it into tensors using complex Morlet wavelets which were finally input into a convolutional neural network (CNN). Experimental results verified that the artificial EEG frame could enhance the performance of the classifier and obtain higher accuracy.

Panwar et al. (2020) proposed a WGAN (Eq. 3) with gradient penalty to synthesize EEG data for rapid serial visual presentation (RSVP) task. It is worth noting that WGAN applied Wasserstein distance to measure the distance between real and generated data.


(3) $$W\left(P_{r},P_{g}\right)=E_{x_{r}∼p_{r}}\left[D\left(x_{r}\right)\right]-E_{x_{g}∼p_{g}}\left[D\left(x_{f}\right)\right]$$

Where *P*_*r*_ and *P*_*g*_ are the distribution of real data *x*_*r*_ and generated data *x*_*g*_. *W* represents the distance of two distributions and *E* is mean value. To improve the training stability and convergence, they utilized a gradient penalty to optimize the training process. Meanwhile, the proposed method addressed the problems of frequency artifacts and instability in the training process of DA. To evaluate the effectiveness of DA, they proposed two evaluation indices (visual inspection and log-likelihood score from Gaussian mixture models) to assess the quality of generated data. Experiments show that presentation-associated patterns of EEG could be seen clearly in generated data and they obtained significant improvement based on the EEGNet model after DA in the RSVP task (Lawhern et al., 2018). A similar method was also performed in Aznan et al. (2019).

Aznan et al. (2019) applied WGAN to generate synthetic EEG data that optimizes the efficiency of interaction in the SSVEP task. After that, they performed generated EEG to the pre-trained classifier in the offline stage and finetune classifier by real-collection EEG. This approach was used to control the robot and achieve real-time navigation. Results show that the DA method significantly improves the accuracy in real-time navigation tasks across multiple subjects.

Yang et al. (2020) deemed that typical DA methods of GT and NI ignored the effect of signal-to-noise ratio (SNR) across trials. Therefore, they proposed a novel DA method by randomly averaging EEG data, which artificially generates EEG data with different SNR patterns. The DA was achieved by randomly taking *n* (1 < *n* < *N*) examples from the same category to calculate the average potential at each iteration, where *N* represents the number of all trials. RNN and CNN were used to classify different specific frequencies in the visual evoked potential (VEP) task and obtained significant improvement after DA.

Li et al. (2019) discussed the effect of noise addition for time series form and spectrums signals in the MI-BCI task, respectively. They applied CNN combined with channel-projection and mixed-scale to classify 4-class MI signals and concluded that noise may destroy the amplitude and phase information of time-series signals, but cannot change the feature distribution of spectrum. Therefore, they performed STFT to transform time series EEG signal into spectral images, which was defined as amplitude-perturbation DA. Results show that the performance has been improved using DA almost for all subjects in two public datasets.

Lee et al. (2020) investigated a novel DA method called borderline-synthetic minority over-sampling technique (Borderline -SMOTE). It generates synthetic data from minority class by using the *m* nearest neighbors from the instance of the minority class and then adding these instances into real data by weighting calculation. The effectiveness of DA was evaluated by EEG data collected from the P300 task. Results show that the proposed methods could enhance the robustness of decision boundaries to improve the classification accuracy of P300 based on BCIs.

Regarding EEG-based passive BCIs, they have gradually become more prominent in research (Zander et al., 2009; Cotrina et al., 2014; Aricò et al., 2018), and are used to detect and monitor the affective states of humans. In this part, we introduce some cases of DA application to passive BCIs.

Kalaganis et al. (2020) proposed a DA method based on graph-empirical mode decomposition (EMD) to generate EEG data, which combines the advantage of multiplex network model and graph variant of classical empirical mode decomposition. They designed a sustained attention driving task in a virtual reality environment, while realizing the automatic detection for the state of humans using graph CNN. The experimental results show that the exploration of the graph structure of EEG signal could reflect the spatial feature of signal and the methodology of integrating graph CNN with DA has obtained a more stable performance.

Wang et al. (2018) discussed the limitations of DA for EEG in emotion recognition tasks and pointed out that the features of EEG in emotion detection tasks have a high correlation with time sequence. However, direct geometric transformations and noise injection may destroy the feature in the time domain, which may cause a negative DA effect. Based on these considerations, they added Gaussian noise to each feature matrix of the original data to obtain new training samples. The calculation could be defined as:


(4) $$P_{G}\left(z\right)=\frac{1}{\sigma \sqrt{2\pi }}e^{-\frac{{\left(z-\mu \right)}^{2}}{2\delta^{2}}}$$

(5) $$x_{g}=x_{r}+x_{r}*P_{G}\left(z\right)$$

Where μ and σ are mean value and standard deviation, respectively, *P* is probability density function, and *z* is Gaussian random variable. *x*_*g*_ is generated data after noise injection. There are three classification models, namely LeNet, ResNet, and SVM, that were used to evaluate the performance. Results show the generated data could significantly improve the performance for the classifier based on LeNet and ResNet. However, it obtains little effect on the SVM model.

Luo et al. (2020) applied conditional Wasserstein GAN (cWGAN) and selective VAE (sVAE) to enhance the performance of the classifier in the emotion recognition tasks. The loss functions of sVAE is defined as follows:


(6) $$\mathrm{maxELBO}=\sum_{i}\sum_{k}\left[{\left(x_{r}-x_{g}\right)}^{2}\right]+\frac{1}{2}\left(\sum \left(x_{r}\right)+\mu^{2}\left(x_{r}\right)-1-log\sum x_{r}\right)$$

Where *ELBO* represent the evidence lower bound and *x*_*r*_ and *x*_*g*_ is real data and generated data, respectively. The goal of optimization was to maximize ELBO which was equal to minimizing the KL divergence between the real data and generated data. Based on the loss function of GAN, an extra penalty term is added to it:


(7) $$\mathrm{minmaxL}\left(x_{r},x_{g}\right)=E_{x_{r}∼x_{r}}\left[D\left(x_{r}\right)\right]-E_{x_{g}∼x_{g}}\left[D\left(x_{g}\right)\right]-\lambda E_{\hat{x_{r}}∼\hat{x_{r}}}\left[\left({∥\nabla_{\hat{x}}D\left(\hat{x}\right)∥}_{2}\right)-1\right]$$

Where λ is weight coefficient for the trade-off between the original objective and gradient penalty, and $\hat{x}$ represents the data points sampled from the straight line between a real distribution and generated distribution. ∥⋅∥_2_ is 2-norm value. In their work, the training samples of DA models were transformed into the forms of power spectral density or differential entropy and the performance of different classifiers are compared after DA. Experiments show that two representations of EEG signals were suitable for the requirement of the artificial datasets that enhances the performance of the classifier.

Bashivan et al. (2015) emphasized the challenge in modeling cognition signals from EEG was extracting the representation of signals across subjects/sessions and suggested that DNN had an excellent ability for feature extraction. Therefore, they transformed the raw EEG signal to topology-preserving multi-spectral images as training sets in a mental load classification task. To address the overfitting and weak generalization ability, they randomly added noise to spectral images to generate training sets. However, this DA method did not significantly improve classification performance, just strengthened the stability of the model.

To comprehensively show the implementation, we summarize the details of the application of DA in EEG decoding in Table 2.

**TABLE 2:** Summary of data augmentation for EEG decoding based on DNNs.

EEG paradigms	Channel of EEG	Subjects	DA methods	Input form of DA	Classifier	Improvement of accuracy after DA	Datasets	References
Driving detection	30	27	EMD	Time-series data	Graph CNN	72–95%	Private dataset (27 subjects)	Kalaganis et al., 2020
SD	23	23	WGAN	Time-series data	CNN	72.11–95.89%	CHB-MIT (PhysioNet, 2010)	Wei et al., 2019
ER	14	18	GAN	Time-series data	DNN	NA to 98.4%	Private dataset (18 subjects)	Chang and Jun, 2019
ER	15/32	NA	NI	Differential entropy	SVM/ResNet	40.8–45.4%	SEED (Chang and Jun, 2019)/MAHNOB-HCI dataset (Zheng and Lu, 2015)	Wang et al., 2018
ER	15/32	15	cWGAN/sVAE	Power spectral density/Differential entropy	SVM/DNN	44.9–90.8%	SEED/DEAP (Chang and Lin, 2011)	Luo et al., 2020
ER	32	15	NI	Time-series data	3D-CNN	79.11–88.49% 79.12–87.44%	DEAP (Koelstra et al., 2012)	Shawky et al., 2018
ER	NA	NA	CycleGAN	Time-series data	CNN	Average improvement of 3.7%∼8%	FER2013/SFEW/JAFFE datasets	Zhu et al., 2018
ERP	31	37	Paired trial	Time-series data	DNN	Average improvement of 20∼30%	ERP datasets from Williams et al. (2020)	Williams et al., 2020
P300	32	44	Borderline-SMOTE	Time-series data	SVM/CNN	Average improvement of 5∼15%	Private dataset (44 subjects)	Lee et al., 2020
MI	14	1	Conditional DCGAN	Time-frequency representation	CNN	78–83%	BCIC II-dataset III (Johnson et al., 2015)	Schlögl, 2003
MI	22/44	9/NA	NI	Spectral image	CP-MixedNet	Average improvement of 1.1∼4.5%	BCIC IV-dataset 2a (Zhang and Liu, 2018) /HGD (Ang et al., 2012)	Li et al., 2019
MI	14	1/5	EMD	Time-series data	CNN/WNN	77.9–82.9% 88.0–90.1%	BCIC II-dataset III/Five subject’s experiments	Zhang Z. et al., 2019
MI	14/3	1/9	GT	Time-frequency representation	CNN	NA	BCIC II-dataset III/BCIC IV-dataset 2b (Leeb et al., 2007; Schirrmeister et al., 2017)	Shovon et al., 2019
MI	3	9	Feature transform	Time-series data	CNN	Average improvement of 5∼10%	BCIC IV-dataset 2b	Huang et al., 2020
MI	3/3	4/9	DCGAN	Spectral image	CNN	74.5–83.2% 80.6–93.2%	BCIC IV-dataset 1 (Huang et al., 2020) /BCIC IV-dataset 2b (BCI Competition, 2008)	Zhang et al., 2020
MI	22	9	NI/GT	Time-series data	LSTM	NA	BCIC IV-dataset 2a	Freer and Yang, 2019
MI	22/3	9/9	SW	Time-series data	RM classifier	NA to 80.4%/82.39%	BCIC IV-dataset 2a/BCIC IV-dataset 2b	Majidov and Whangbo, 2019
MI	62	14	Conditional DCGAN	Spectral image	CNN	Average improvement of 3.22∼5.45%	Private dataset (14 subjects)	Fahimi et al., 2020
MI	22/3	9/9	Feature transform	Time-series data	HS-CNN	85.6–87.6%	BCIC IV-dataset 2a/BCIC IV-dataset 2b	Dai et al., 2019
MI	22/60	9/3	Extended CSSP	Feature matrix	FLDA	Average improvement of 0.3∼31.2%	BCIC IV-dataset 2a/BCIC III -dataset IIIa (Blankertz and BCI Competition, 2005)	Gubert et al., 2020
RSVP	256	10	WGAN	Time-series data	EEGNet	NA	BCIT X2 dataset (Dong et al., 2016)	Lawhern et al., 2018
Sleep stage classification	2/2	20/25	Oversampling model	Time-series data	BLSTM	Average improvement of 0.1∼2.0%	SA dataset/Sleep-EDF database (Goldberger et al., 2000)	Sun et al., 2019
SSVEP	32	8	Randomly average	Time-series data	RNN	Average improvement of 3∼13%	Private dataset (8 subjects)	Yang et al., 2020
MW detection	4	8	NI	Time-series data	DBN	NA	Private dataset (8 subjects)	Yin and Zhang, 2017b
MW detection	64	15	NI	Spectral image	Multi-frame classifier	NA	Private dataset (8 subjects)	Bashivan et al., 2014, 2015
MW detection	11	7	NI	Spectral image	SAE	34.2–75%	Private dataset (7 subjects)	Yin and Zhang, 2017a
MW detection	64	22	NI	Spectral image	RNN + CNN	NA to 93%	Private dataset (22 subjects)	Kuanar et al., 2018
MI	3	5	NI	Time-series data	CNN	NA	BCIC IV-dataset 2b	Parvan et al., 2019
MW detection	1	30	GAN	Spectral image	Boast	90–95%	NA	Piplani et al., 2018
MI	1	1	GAN	Spectral image	Distance measurement	NA	NA	Hartmann et al., 2018
ER	32	32	GAN	Spectral image	CWGAN	Average improvement of 3∼20%	SEED	Luo and Lu, 2018
MI	3	1	GAN	Spectral image	CNN	77–79%	BCIC IV-dataset 2b	Zhang X. et al., 2018
ER	14	18	GAN	Time-series data	GANA	97–98%	CHB-MIT	Chang and Jun, 2019
MI	3	9	GAN	Time-series data	CNN + LSTM	NA to 76%	BCIC IV-dataset 2b	Yang et al., 2019
RSVP	256	10	GAN	Time-series data	CNN	Average improvement of 0.7∼2%	BCIT X2 RSVEP Dataset (Touryan et al., 2014)	Panwar et al., 2019
SD	100	18	SW	Spatio-temporal signal	CNN	NA to 97%	Clinic dataset (100 subjects)	O’Shea et al., 2017
SD	100	10	SW	Time-series data	CNN	NA	University of Bonn Dataset (Andrzejak et al., 2001)	Ullah et al., 2018
SD	16	2	SW	Spectral image	CNN	NA	Clinic dataset	Truong et al., 2018
SSC	4	NA	SW	Time-series data	CNN	82.9–85.7%	Sleep-EDF database	Mousavi et al., 2019
SD	18	29	SW	Time-series data	CNN	NA to 93%	Clinic dataset	Avcu et al., 2019
MI	3	9	SW	Spectral image	CNN	NA to 84%	BCIC IV-dataset 2b	Tayeb et al., 2019
SD	18	24	SW	Spatio-temporal signal	LSTM	70–78%	CHB-MIT	Tsiouris et al., 2018
SD	NA	2	SW	Time-series data	CNN	NA	Clinic dataset	Tang et al., 2017
RSVP	64	12	Oversampling model	Time-series data	CNN	83.99–86.96%	Private dataset (12 subjects)	Manor and Geva, 2015
Movement of eye	2	NA	Oversampling model	Feature matrix	MLP	NA to 82%	MAHNOB HCI-Tagging database (Soleymani et al., 2012)	Drouin-Picaro and Falk, 2016
SSC	20	62	Oversampling model	Time-series data	CNN	Average improvement of 1.7∼20%	Sleep-EDF	Supratak et al., 2017
SSC	20	62	Oversampling model	Time-frequency representation	Mixer networks	89.3–90.1%	NA	Dong et al., 2017
SSC	14	121	Oversampling model	Spectral image	CNN	NA	Private dataset (121 subjects)	Ruffini et al., 2018
SD	23	23	Oversampling model	Spatio-temporal signal	CNN + LSTM	NA	CHB-MIT	Thodoroff et al., 2016
SSC	NA	NA	Oversampling model	Spectral image	CNN	NA to 99%	Private dataset	Sengur et al., 2019
SSC	16	NA	Feature transform	Time-series data	CNN	72–75%	Private dataset	Schwabedal et al., 2018
ER	32	32	GT	Wavelet feature	SAE	NA to 68.75%	DEAP	Surrogates Said et al., 2017
ER	32	22	GT	Feature matrix	NN	40.8–45.4%	DEAP	Frydenlund and Rudzicz, 2015
SSC	19	155	Repeat sampling	Entropy feature	CNN	NA to 81.4%	Clinic dataset	Deiss et al., 2018
ER	32	32	GT	Wavelet feature	CNN	Average improvement of 2.2∼5%	DEAP	Mokatren et al., 2019
MI	3/22	9/9	NI	Time-series data	Inception NN	Average improvement of 3%	BCIC IV-dataset 2b BCIC IV-dataset 2a	Zhang et al., 2021

*Abbreviations: BCIC, BCI Competition; BLSTM, Bidirectional Long Short-Term Memory network; CSSP, Common Spectral Spatial Patterns; DBN, Deep Belief Networks; EMD, Empirical Model Decomposition; ER, Emotion recognition; FLDA, Fisher Linear Discriminant Analysis; HS-CNN, A CNN with Hybrid Convolution Scale; MW, Mental Workload; MLP, Multi-Layer Perception; LeNet, Deep neural network proposed by Lecun and Bottou (1998); NA, Not Applicable; NN, Neural Network; KL, Kullback-Leibler; WNN, Wavelet Neural Network; RM, Riemannian Manifold; ReLU, Rectified Linear Units; SVM, Support Vector Machines; SAE, Stacked Autoencoder; SW, Sliding Window; SD, Seizure Detection; SSC, Sleep Stage Classification; ResNet, Deep neural network proposed by He et al. (2016).*

## Discussion

The limitation of small-scale datasets hinders the application of DL for EEG classification. Recently, the strategy of DA has received widespread attention and is employed to improve the performance of DNNs. However, there remain several issues worth discussing.

Taking the above discussion into consideration, we found that the input forms of DA models could be divided into three categories: time-series data, spectral image, and feature matrix. We also found that researchers preferred to convert EEG signals into image signals for subsequent processing in MI tasks. One possible reason might be that the features of MI are often accompanied by changes in frequency band energy, i.e., event-related desynchronization (ERD)/event-related synchronization (ERS; Phothisonothai and Nakagawa, 2008; Balconi and Mazza, 2009). This phenomenon indicated that more significant feature representations of MI-EEG were displayed in the time-frequency space rather than the time domain. While the EEG based on VEP paradigms prefer to employ the time-series signal as the input, which has the strict requirement of being time-locked and contains more obvious features in time sequence (Basar et al., 1995; Kolev and Schurmann, 2009; Meng et al., 2014). Another form of input is the feature matrix that could be extracted by wavelet, entropy, STFT, power spectral density, and so on (Subasi, 2007; Filippo et al., 2009; Seitsonen et al., 2010; Lu et al., 2017; Lashgari et al., 2020).

From a difference of implementation point of view, DA can be divided into input space augmentation and feature space augmentation. Indeed, the former aspect has the advantage of interpretability and takes lower computational costs. However, we found that the operation in feature space could obtain more significant improvements than in input space based on the results of classification performance presented in Table 2. One explanation is that this type of DA model could extract an intrinsic representation of data due to the incredible ability of non-linear mapping and automatic feature extraction.

Generative adversarial networks have become popular for generating EEG signals in recent years (Hung and Gan, 2021), although it has still not been clearly demonstrated to be the most effective strategy across different EEG tasks. Due to the limited number of studies, it is still unclear which method is the more popular technique. Consequently, researchers should select the appropriate DA method according to the paradigm type and feature representation of EEG.

Previous studies show that DA could improve the decoding accuracy of EEG to varying degrees in different EEG tasks. However, this improvement varies greatly in different data sets and preprocessing modes. There are several possible explanations to be discussed. First, most studies have not discussed whether DA produces negative effects in the training stage of the classifier. As mentioned in the above discussion, EEG signals are accompanied by strong noise and multi-scale artifact. But existing DA methods are global operations, which cannot effectively distinguish these irrelevant components. Meanwhile, EEG signals collected from specific BCI tasks (SSVEP, P300) perform features that are time-locked and phase-locked, which may cause wrong feature representation using GT to produce artificial data. While GT performs effectively in MI and ER tasks due to this kind of signal having no strict requirement for feature-locking. Therefore, feature representation of EEG should be analyzed before the application of GT. Second, there are a few studies that discuss the boundary conditions of the feature distribution for generated data, even though it is one of the important guarantees of data validity.

Another important issue worthy to discuss is how much generated data could most effectively enhance the performance of a classifier. Researchers have explored the influence of different ratios of real data (RD) and generated data (GD) for classification performance and demonstrated that the enhancement effect does not increase with the size of GD (Zhang et al., 2020). Research on the effect of different amounts of training data to the classification performance using artificial data has indicated that the improvement of performance requires at least a doubling size of GD (Zhang and Liu, 2018). Consequently, the size of the GD should be determined by multi-group trials with different mix proportion.

Based on the above analysis we believe that the following studies are worthy of exploring in further research. First, different DA methods can be combined to extend datasets and augmentation would be executed both in input space and feature space. For example, generated data based on GT can be put into GANs to realize secondary augmentation, which may improve the diversity of generated data. Second, combining meta-learning with data enhancement might reveal why DA affects classification tasks, which may improve the interpretability of generated data. Meanwhile, DA based on GAN is a mainstream method at present, but how to improve the quality of generated data is still a valuable point.

## Conclusion

Collecting large-scale EEG datasets is a difficult task due to the limitations of available subjects, experiment time, and operation complexity. Data augmentation has proven to be a promising approach to avoid overfitting and improve the performance of DNNs. Consequently, the research state of DA for EEG decoding based on DNNs is discussed in the study. The latest studies in the past 5 years have been discussed and analyzed in this work. Based on the analysis of their results, we could conclude that DA is able to effectively improve the performance in EEG decoding tasks. This review presents the current practical suggestions and performance outcomes. It may provide guidance and help for EEG research and assist the field to produce high-quality, reproducible results.

## Author Contributions

CH is responsible for manuscript writing. JL draw related figure. YZ and WD guide the literature collection and structure of the manuscript. All authors contributed to the article and approved the submitted version.

## Conflict of Interest

CH and JL were employed by the company Shenzhen EEGSmart Technology Co., Ltd. The remaining authors declare that the research was conducted in the absence of any commercial or financial relationships that could be construed as a potential conflict of interest.

## Publisher’s Note

All claims expressed in this article are solely those of the authors and do not necessarily represent those of their affiliated organizations, or those of the publisher, the editors and the reviewers. Any product that may be evaluated in this article, or claim that may be made by its manufacturer, is not guaranteed or endorsed by the publisher.

## References

[B1] Abdelfattah S. M. Abdelrahman G. M. Wang M. (2018). “Augmenting the size of EEG datasets using generative adversarial networks,” in *Proceedings of the 2018 IEEE International Joint Conference on Neural Networks (IJCNN)*, Rio, 1–6. 10.1109/IJCNN.2018.8489727

[B2] Andrzejak R. G. Lehnertz K. Mormann F. Rieke C. David P. Elger C. E. (2001). . Indications of nonlinear deterministic and finite-dimensional structures in time series of brain electrical activity: dependence on recording region and brain state. *Phys. Rev. E Stat. Nonlin. Soft Matter Phys.* 64:061907. 10.1103/PhysRevE.64.061907 11736210

[B3] Ang K. K. Chin Z. Y. Wang C. Guan C. Zhang H. (2012). Filter bank common spatial pattern algorithm on BCI competition IV datasets 2a and 2b. *Front. Neurosci.* 6:39. 10.3389/fnins.2012.00039 22479236 PMC3314883

[B4] Arico P. Borghini G. Di Flumeri G. Campagne A. (2017). Passive BCI in operational environments: insights, recent advances and future trends. *IEEE Trans. Biomed. Eng.* 64 1431–1436. 10.1109/TBME.2017.2694856 28436837

[B5] Aricò P. Gianluca B. Gianluca D. F. Nicolina S. Fabio B. (2018). Passive BCI beyond the lab: current trends and future directions. *Physiol. Meas.* 39:08TR02. 10.1088/1361-6579/aad57e 30039806

[B6] Arslan M. Güzel M. Demirci M. Ozdemir S. (2019). “SMOTE and gaussian noise based sensor data augmentation,” in *Proceedings of the 4th International Conference on Computer Science and Engineering (UBMK)*, Samsun. 10.1109/UBMK.2019.8907003

[B7] Atagün M. I. (2016). Brain oscillations in bipolar disorder and lithium-induced changes. *Neuropsychiatr. Dis. Treat.* 12 589–601. 10.2147/NDT.S100597 27022264 PMC4788370

[B8] Avcu M. T. Zhang Z. Chan D. W. S. (2019). “Seizure detection using least Eeg channels by deep convolutional neural network,” in *Proceedings of the ICASSP 2019-2019 IEEE International Conference on Acoustics, Speech and Signal Processing (ICASSP)*, (Piscataway, NJ: IEEE). 10.1109/ICASSP.2019.8683229

[B9] Aznan N. K. N. Connolly J. D. Al Moubayed N. Breckon T. P. (2019). “Using variable natural environment brain-computer interface stimuli for real-time humanoid robot navigation,” in *Proceedings of the 2019 International Conference on Robotics and Automation (ICRA)*, (Piscataway, NJ: IEEE). 10.1109/ICRA.2019.8794060

[B10] Balconi M. Mazza G. (2009). Brain oscillations and BIS/BAS (behavioral inhibition/activation system) effects on processing masked emotional cues. ERS/ERD and coherence measures of alpha band. *Int. J. Psychophysiol.* 74 158–165. 10.1016/j.ijpsycho.2009.08.006 19709636

[B11] Basar E. Basar-Eroglu C. Demiralp T. Schurmann M. (1995). Time and frequency analysis of the brain’s distributed gamma-band system. *Eng. Med. Biol. Mag.* 14 400–410. 10.1109/51.395322

[B12] Bashivan P. Bidelman G. M. Yeasin M. (2014). Spectro temporal dynamics of the EEG during working memory encoding and maintenance predicts individual behavioral capacity. *Eur. J. Neurosci.* 40 3774–3784. 10.1111/ejn.12749 25288492

[B13] Bashivan P. Rish I. Yeasin M. Codella N. (2015). Learning representations from EEG with deep recurrent-convolutional neural networks. *arXiv* [Preprint] arXiv:1511.06448

[B14] BCI Competition (2008). *Graz Data Sets 2A and 2B.* Available online: http://www.bbci.de/competition/iv/ (accessed May 30, 2019)

[B15] Bengio Y. Courville A. Vincent P. (2013). Representation learning: a review and new perspectives. *IEEE Trans. Pattern Anal. Mach. Intell*. 35, 1798–1828. 10.1109/TPAMI.2013.50 23787338

[B16] Blankertz B. BCI Competition (2005). *Dataset IIIA, 2018.* Available online at: http://www.bbci.de/competition/iii/ (accessed September 25).

[B17] Boernama A. Setiawan N. A. Wahyunggoro O. (2021). “Multiclass classification of brain-computer interface motor imagery system: a systematic literature review,” in *Proceedings of the 2021 International Conference on Artificial Intelligence and Mechatronics Systems (AIMS)*, Bandung. 10.1109/AIMS52415.2021.9466056

[B18] Bonassi G. Biggio M. Bisio A. Ruggeri P. Bove M. Acanzino L. (2017). Provision of somatosensory inputs during motor imagery enhances learning-induced plasticity in human motor cortex. *Sci. Rep.* 7:9300. 10.1038/s41598-017-09597-0 28839226 PMC5571213

[B19] Chang C. C. Lin C. J. (2011). LIBSVM: a library for support vector machines. *ACM Trans. Intell. Syst. Technol.* 2:27. 10.1145/1961189.1961199

[B20] Chang S. Jun H. (2019). Hybrid deep-learning model to recognize emotional responses of users towards architectural design alternatives. *J. Asian Arch. Build. Eng.* 18 381–391. 10.1080/13467581.2019.1660663

[B21] Cotrina A. Castillo J. Bastos T. (2014). “Towards an architecture of a hybrid BCI based on SSVEP-BCI and passive-BCI,” in *Proceedings of the 36th Annual International Conference of the IEEE Engineering in Medicine and Biology Society (EMBC’14)*, Chicago, IL. 10.1109/EMBC.2014.6943847 25570215

[B22] Cui X. Goel V. Kingsbury B. (2014). “Data augmentation for deep convolutional neural network acoustic modeling,” in *Proceedings of the 2015 IEEE International Conference on Acoustics, Speech and Signal Processing (ICASSP)*, Piscataway, NJ: IEEE. 10.1109/ICASSP.2015.7178831

[B23] Cui X. Goel V. Kingsbury B. (2015). Data augmentation for deep neural network acoustic modeling. *IEEE/ACM Trans. Audio Speech Lang. Process.* 23 1469–1477. 10.1109/TASLP.2015.2438544 PMC4784988 26973851

[B24] Dai G. Zhou J. Huang J. Wang N. (2019). HS-CNN: a CNN with hybrid convolution scale for EEG motor imagery classification. *J. Neural Eng.* 17:016025. 10.1088/1741-2552/ab405f 31476743

[B25] Deiss O. Biswal S. Jin J. Sun H. Westover M. B. Sun J. (2018). HAMLET: interpretable human and machine Co-LEarning technique. *arXiv* [Preprint] arXiv:1803.09702

[B26] Deng J. Zhang Z. Eyben F. Schuller B. (2014). Autoencoder-based unsupervised domain adaptation for speech emotion recognition. *IEEE Signal Process. Lett.* 21 1068–1072. 10.1109/LSP.2014.2324759

[B27] Dong C. Loy C. C. He K. Tang X. (2016). Image super-resolution sing deep convolutional networks. *IEEE Trans. Pattern Anal. Mach. Intell.* 38 295–307. 10.1109/TPAMI.2015.2439281 26761735

[B28] Dong H. Supratak A. Pan W. Wu C. Matthews P. M. Guo Y. (2017). Mixed neural network approach for temporal sleep stage classification. *IEEE Trans. Neural Syst. Rehabil. Eng.* 26 324–333. 10.1109/TNSRE.2017.2733220 28767373

[B29] Drouin-Picaro A. Falk T. H. (2016). “Using deep neural networks for natural saccade classification from electroencephalograms,” in *Proceedings of the 2016 IEEE EMBS International Student Conference (ISC)*, Ottawa, ON. 10.1109/EMBSISC.2016.7508606

[B30] Fahimi F. Dosen S. Ang K. K. Mrachacz-Kersting N. Guan C. (2020). Generative adversarial networks-based data augmentation for brain-computer interface. *IEEE Trans. Neural Netw. Learn. Syst.* 32 4039–4051. 10.1109/TNNLS.2020.3016666 32841127

[B31] Filippo C. Melissa Z. Laura A. Fabio B. Mauro U. (2009). Changes in EEG power spectral density and cortical connectivity in healthy and tetraplegic patients during a motor imagery task. *Comput. Intell. Neurosci.* 2009:279515. 10.1155/2009/279515 19584939 PMC2703829

[B32] Freer D. Yang G. Z. (2019). Data augmentation for self-paced motor imagery classification with C-LSTM. *J. Neural Eng.* 17 016041. 10.1088/1741-2552/ab57c0 31726440

[B33] Frydenlund A. Rudzicz F. (2015). “Emotional affect estimation using video and EEG data in deep neural networks,” in *Proceedings of the Canadian Conference on Artificial Intelligence*, (Cham: Springer). 10.1007/978-3-319-18356-5_24

[B34] Goldberger A. L. Amaral L. A. Glass L. Hausdorff J. M. Ivanov P. C. Mark R. G. (2000). Physiobank, physiotoolkit, and physionet. *Circulation* 101 215–220. 10.1161/01.CIR.101.23.e215 10851218

[B35] Goodfellow I. Pouget-Abadie J. Mirza M. Xu B. Warde-Farely D. Ozair S. (2014). “Generative Adversarial Nets,” in *Proceedings of the International Conference on Neural Information Processing Systems*, Montreal, QC, 2672–2680.

[B36] Gubert P. H. Costa M. H. Silva C. D. Trofino-Neto A. (2020). The performance impact of data augmentation in CSP-based motor-imagery systems for BCI applications. *Biomed. Signal Process. Control* 62 102152. 10.1016/j.bspc.2020.102152

[B37] Hartmann K. G. Schirrmeister R. T. Ball T. (2018). EEG-GAN: generative adversarial networks for electroencephalograhic (EEG) brain signals. *arXiv* [Preprint] arXiv:1806.01875

[B38] He K. Zhang X. Ren S. Sun J. (2016). “Deep residual learning for image recognition,” in *Proceedings of the IEEE Conference on Computer Vision and Pattern Recognition*, Las Vegas, NV, 770–778. 10.1109/CVPR.2016.90

[B39] Heyne L. Rhensius J. Cho Y. J. Bedau D. Krzyk S. Dette C. (2009). Geometry-dependent scaling of critical current densities for current-induced domain wall motion and transformations. *Phys. Rev.* 80 184405.1–184405.4. 10.1103/PhysRevB.80.184405

[B40] Hinton G. Deng L. Yu D. Yu D. Dahl G. E. Kingsbury B. (2012). Deep neural networks for acoustic modeling in speech recognition: the shared views of four research groups. *IEEE Signal Process. Mag.* 29 82–97. 10.1109/MSP.2012.2205597

[B41] Huang W. Wang L. Yan Z. Yanjun L. (2020). “Classify motor imagery by a novel CNN with data augmentation*,” in *Proceedings of the 2020 42nd Annual International Conference of the IEEE Engineering in Medicine and Biology Society (EMBC) in conjunction with the 43rd Annual Conference of the Canadian Medical and Biological Engineering Society*, Montreal, QC. 10.1109/EMBC44109.2020.9176361 33017962

[B42] Hung S. K. Gan J. Q. (2021). “Augmentation of small training data using GANs for enhancing the performance of image classification,” in *Proceedings of the 2020 25th International Conference on Pattern Recognition (ICPR)*, Milan. 10.1109/ICPR48806.2021.9412399

[B43] Johnson M. R. McCarthy G. Muller K. A. Brudner S. N. Johnson M. K. (2015). Electrophysiological correlates of refreshing: event-related potentials associated with directing reflective attention to face, scene, or word representations. *J. Cogn. Neurosis.* 27 1823–1839. 10.1162/jocn_a_00823 PMC4796063 25961640

[B44] Kalaganis F. P. Laskaris N. A. Chatzilari E. Nikolopoulos S. Kompatsiaris I. Y. (2020). A data augmentation scheme for geometric deep learning in personalized brain–computer interfaces. *IEEE Access* 8 162218–162229. 10.1109/ACCESS.2020.3021580

[B45] Kaneko T. Kameoka H. Tanaka K. Hojo N. (2019). CycleGAN-VC2: improved CycleGAN-based non-parallel voice conversion. *arXiv* [Preprint] arXiv:1904.04631 [cs.SD] 10.1109/ICASSP.2019.8682897

[B46] Koelstra S. Mühl C. Soleymani M. Lee J.-S. Yazdani A. Ebrahimi T. (2012). DEAP: a database for emotion analysis; using physiological signals. *IEEE Trans. Affect. Comput.* 3 18–31. 10.1109/T-AFFC.2011.15

[B47] Kolev V. Schurmann M. (2009). Event-related prolongation of induced Eeg rhythmicities in experiments with a cognitive task. *Int. J. Neurosci.* 67 199–213. 10.3109/00207459208994785 1305635

[B48] Kuanar S. Athitsos V. Pradhan N. Mishra A. Rao K. R. (2018). “Cognitive analysis of working memory load from EEG, by a deep recurrent neural network,” in *Proceedings of the 2018 IEEE International Conference on Acoustics, Speech and Signal Processing (ICASSP)*, Calgary, AB. 10.1109/ICASSP.2018.8462243

[B49] Lashgari E. Liang D. Maoz U. (2020). Data augmentation for deep-learning-based electroencephalography. *J. Neurosci. Methods* 346:108885. 10.1016/j.jneumeth.2020.108885 32745492

[B50] Lawhern V. J. Solon A. J. Waytowich N. R. Gordon S. M. Hung C. P. Lance B. J. (2018). EEGNet: a compact convolutional neural network for EEG-based brain- computer interfaces. *J. Neural Eng.* 15:056013. 10.1088/1741-2552/aace8c 29932424

[B51] Lecun Y. Bottou L. (1998). Gradient-based learning applied to document recognition. *Proc. IEEE* 86 2278–2324. 10.1109/5.726791

[B52] Lee T. Kim M. Kim S. P. (2020). “Data augmentation effects using borderline-SMOTE on classification of a P300-based BCI,” in *Proceedings of the 2020 8th International Winter Conference on Brain-Computer Interface (BCI)*, Gangwon. 10.1109/BCI48061.2020.9061656

[B53] Leeb R. Lee F. Keinrath C. Scherer R. Bischof H. Pfurtscheller G. (2007). Brain–computer communication: motivation, aim, and impact of exploring a virtual apartment. *IEEE Trans. Neural Syst. Rehabil. Eng.* 15 473–482. 10.1109/TNSRE.2007.906956 18198704

[B54] Levitskaya O. S. Lebedev M. A. (2016). Brain-computer interface: the future in the present. *Bull. Russ. State Med. Univ.* 2, 4–15. 10.24075/brsmu.2016-02-01

[B55] Li Y. Zhang X. R. Zhang B. Lei M. Y. Cui W. G. Guo Y. Z. (2019). A channel-projection mixed-scale convolutional neural network for motor imagery EEG decoding. *IEEE Trans. Neural Syst. Rehabil. Eng.* 27 1170–1180. 10.1109/TNSRE.2019.2915621 31071048

[B56] Liberati A. Altman D. G. Tetzlaff J. Mulrow C. Gøtzsche P. C. Ioannidis J. (2009). The PRISMA statement for reporting systematic reviews and meta-analyses of studies that evaluate health care interventions: explanation and elaboration. *Epidemiol. Biostat. Public Health* 6 e1–e34. 10.1016/j.jclinepi.2009.06.006 19631507

[B57] Lotte F. Bougrain L. Cichocki A. Clerc M. Congedo M. Rakotomamonjy A. (2018). A review of classification algorithms for EEG-based brain-computer interfaces: a 10-year update. *J. Neural Eng.* 15:031005. 10.1088/1741-2552/aab2f2 29488902

[B58] Lotte F. Congedo M. Lécuyer A. Lamarche F. (2007). A review of classification algorithms for EEG-based brain–computer interfaces. *J. Neural Eng.* 4 R1–R13. 10.1088/1741-2560/4/2/R01 17409472

[B59] Lu Y. Jiang H. Fabio B. Mauro U. (2017). “Classification of EEG signal by STFT-CNN framework: identification of right-/left-hand motor imagination in BCI systems,” in *Proceedings of the 7th International Conference on Computer Engineering and Networks*, Shanghai. 10.22323/1.299.0001

[B60] Luck S. J. (2005). *An Introduction to The Event-Related Potential Technique.* Rijeka: Sveučilište u Rijeci.

[B61] Luo Y. Lu B.-L. (2018). “EEG data augmentation for emotion recognition using a conditional Wasserstein GAN,” in *Proceedings of the 2018 40th Annual International Conference of the IEEE Engineering in Medicine and Biology Society (EMBC)*, (Piscataway, NJ: IEEE). 10.1109/EMBC.2018.8512865 30440924

[B62] Luo Y. Zhu L. Z. Wan Z. Y. Lu B. L. (2020). Data augmentation for enhancing EEG-based emotion recognition with deep generative models. *J. Neural Eng.* 17:056021. 10.1088/1741-2552/abb580 33052888

[B63] Majidov I. Whangbo T. (2019). Efficient classification of motor imagery electroencephalography signals using deep learning methods. *Sensors* 19:1736. 10.3390/s19071736 30978978 PMC6479542

[B64] Manor R. Geva A. B. (2015). Convolutional neural network for multi-category rapid serial visual presentation BCI. *Front. Comput. Neurosci.* 9:146. 10.3389/fncom.2015.00146 26696875 PMC4667102

[B65] Meng J. Meriño L. M. Robbins K. Huang Y. (2014). Classification of imperfectly time-locked image RSVP events with EEG device. *Neuroinformatics* 12 261–275. 10.1007/s12021-013-9203-4 24037139

[B66] Mokatren L. S. Ansari R. Cetin A. E. Leow A. D. Vural F. Y. (2019). Improved EEG classification by factoring in sensor topography. *arXiv* [Preprint] arXiv:1905.09472

[B67] Mousavi Z. Yousefi Rezaii T. Sheykhivand S. Farzamnia A. Razavi S. N. (2019). Deep convolutional neural network for classification of sleep stages from single-channel EEG signals. *J. Neurosci. Methods* 324:108312. 10.1016/j.jneumeth.2019.108312 31201824

[B68] Nguyen A. Yosinski J. Clune J. (2015). “Deep neural networks are easily fooled: high confidence predictions for unrecognizable images,” in *Proceedings of the 2015 IEEE Conference on Computer Vision and Pattern Recognition (CVPR)*, Boston, MA. 10.1109/CVPR.2015.7298640

[B69] Nicolas-Alonso L. F. Gomez-Gil J. (2012). Brain computer interfaces, a review. *Sensors* 12 1211–1279. 10.3390/s120201211 22438708 PMC3304110

[B70] Okafor E. Smit R. Schomaker L. Wiering M. (2017). “Operational data augmentation in classifying single aerial images of animals,” in *Proceedings of the IEEE International Conference on Innovations in Intelligent SysTems and Applications (INISTA)*, Gdynia. 10.1109/INISTA.2017.8001185

[B71] O’Shea A. Lightbody G. Boylan G. Temko A. (2017). “Neonatal seizure detection using convolutional neural networks,” in *Proceedings of the 2017 IEEE 27th International Workshop on Machine Learning for Signal Processing (MLSP*, Tokyo. 10.1109/MLSP.2017.8168193

[B72] Panwar S. Rad P. Jung T.-P. Huang Y. (2020). Modeling EEG data distribution with a wasserstein generative adversarial network to predict RSVP events. *IEEE Trans. Neural Syst. Rehabil. Eng.* 28 1720–1730. 10.1109/TNSRE.2020.3006180 32746311

[B73] Panwar S. Rad P. Quarles J. Huang Y. (2019). “Generating EEG signals of an RSVP experiment by a class conditioned wasserstein generative adversarial network,” in *Proceedings of the 2019 IEEE International Conference on Systems, Man and Cybernetics (SMC)*, Bari. 10.1109/SMC.2019.8914492

[B74] Parvan M. Athitsos V. Pradhan N. Mishra A. Rao K. R. (2019). “Transfer learning based motor imagery classification using convolutional neural networks,” in *Proceedings of the 2019 27th Iranian Conference on Electrical Engineering (ICEE)*, Yazd. 10.1109/IranianCEE.2019.8786636

[B75] Paschali M. Simson W. Roy A. G. Naeem M. F. Göbl R. Wachinger C. (2019). Data augmentation with manifold exploring geometric transformations for increased performance and robustness. *arXiv* [Preprint] arXiv:1901.04420 [cs.LG] 10.1007/978-3-030-20351-1_40

[B76] Pfurtscheller G. (2000). Spatiotemporal ERD/ERS patterns during voluntary movement and motor imagery. *Elsevier Health Sci.* 53 196–198. 10.1016/S1567-424X(09)70157-6 12740996

[B77] Phothisonothai M. Nakagawa M. (2008). EEG-based classification of motor imagery tasks using fractal dimension and neural network for brain-computer interface. *IEICE Trans. Inf. Syst.* 91 44–53. 10.1093/ietisy/e91-d.1.44

[B78] PhysioNet (2010). *CHB-MIT Scalp EEG* x*Database.* Available online at: https://www.physionet.org/content/chbmit/1.0.0/

[B79] Piplani T. Merrill N. Chuang J. (2018). “Faking it, making it: fooling and improving brain-based authentication with generative adversarial networks,” in *Proceedings of the Biometrics, Theory, Applications and Systems (BTAS ‘18)*, Redondo Beach, CA. 10.1109/BTAS.2018.8698606

[B80] Radford A. Metz L. Chintala S. (2015). Unsupervised representation learning with deep convolutional generative adversarial networks. *arXiv* [Preprint] arXiv:1511.06434

[B81] Rao R. P. (2013). *Brain-Computer Interfacing: An Introduction.* New York, NY: Cambridge University Press. 10.1017/CBO9781139032803

[B82] Regmi K. Borji A. (2018). “Cross-view image synthesis using conditional GANs,” in *Proceedings of the 2018 IEEE/CVF Conference on Computer Vision and Pattern Recognition (CVPR)*, Salt Lake City, UT. 10.1109/CVPR.2018.00369

[B83] Roy R. N. Bonnet S. Charbonnier S. Campagne A. (2013). Mental fatigue and working memory load estimation: interaction and implications for EEG based passive BCI. *Annu. Int. Conf. IEEE Eng. Med. Biol. Soc.* 2013 6607–6610. 10.1109/EMBC.2013.6611070 24111257

[B84] Ruffini G. Ibañez D. Kroupi E. Gagnon J.-F. Soria-Frisch A. (2018). Deep learning using EEG spectrograms for prognosis in idiopathic rapid eye movement behavior disorder (RBD). *bioRxiv* [Preprint] 10.1101/240267

[B85] Schirrmeister R. T. Springenberg J. T. Fiederer L. Glasstetter M. Eggensperger K. Tangermann M. (2017). Deep learning with convolutional neural networks for EEG decoding and visualization. *Hum. Brain Mapp.* 38 5391–5420. 10.1002/hbm.23730 28782865 PMC5655781

[B86] Schlögl A. (2003). *Outcome of the BCI-Competition 2003 on the Graz DataSet.* Berlin: Graz University of Technology.

[B87] Schwabedal J. T. Snyder J. C. Cakmak A. Nemati S. Clifford G. D. (2018). Addressing class imbalance in classification problems of noisy signals by using fourier transform surrogates. *arXiv* [Preprint] arXiv:1806.08675

[B88] Seitsonen E. R. J. Korhonen I. K. J. Gils M. J. V. Huiku M. Lötjönen J. M. P. Korttila K. T. (2010). EEG spectral entropy, heart rate, photoplethysmography and motor responses to skin incision during sevoflurane anesthesia. *Acta Anaesthesiol. Scand.* 49 284–292. 10.1111/j.1399-6576.2005.00654.x 15752389

[B89] Sengur A. Bajaj V. Karabatak M. Tanyildizi E. (2019). “Neutrosophic similarity score-based entropy measure for focal and nonfocal electroencephalogram signal classification,” in *Neutrosophic Set in Medical Image Analysis* (Amsterdam: Elsevier), 247–268. 10.1016/B978-0-12-818148-5.00012-6

[B90] Shawky E. El-Khoribi R. Shoman M. A. I. Wahby M. A. (2018). EEG-based emotion recognition using 3D convolutional neural networks. *Int. J. Adv. Comput. Appl.* 9:329.

[B91] Shorten C. Khoshgoftaar T. M. (2019). A survey on image data augmentation for deep learning. *J. Big Data* 6 1–48. 10.1186/s40537-019-0197-0 PMC8287113 34306963

[B92] Shovon T. H. Nazi Z. A. Dash S. Hossain F. (2019). “Classification of motor imagery EEG signals with multi-input convolutional neural network by augmenting STFT,” in *Proceedings of the 5th International Conference on Advances in Electrical Engineering (ICAEE)*, Dhaka. 10.1109/ICAEE48663.2019.8975578

[B93] Soleymani M. Lichtenauer J. Pun T. Pantic M. (2012). A multimodal database for affect recognition and implicit tagging. *Affect. Comput. IEEE Trans.* 3 42–55. 10.1109/T-AFFC.2011.25

[B94] Subasi A. (2007). EEG signal classification using wavelet feature extraction and a mixture of expert model. *Expert Syst. Appl.* 32 1084–1093. 10.1016/j.eswa.2006.02.005

[B95] Sun C. Fan J. Chen C. Li W. Chen W. (2019). A two-stage neural network for sleep stage classification based on feature learning, sequence learning, and data augmentation. *IEEE Access* 7 109386–109397. 10.1109/ACCESS.2019.2933814

[B96] Supratak A. Hao D. Chao W. Guo Y. (2017). DeepSleepNet: a model for automatic sleep stage scoring based on raw single-channel EEG. *IEEE Trans. Neural Syst. Rehabil. Eng.* 25 1998–2008. 10.1109/TNSRE.2017.2721116 28678710

[B97] Surrogates Said A. B. Mohamed A. Elfouly T. Harras K. Wang Z. J. (2017). “Multimodal deep learning approach for joint EEG-EMG data compression and classification,” in *Proceedings of the 2017 IEEE Wireless Communications and Networking Conference (WCNC)*, San Francisco, CA. 10.1109/WCNC.2017.7925709

[B98] Tang Y. Wada S. Yoshihara K. (2017). “Failure prediction with adaptive multi-scale sampling and activation pattern regularization,” in *2017 IEEE International Conference on Data Mining Workshops (ICDMW)*, New Orleans, LA. 10.1109/ICDMW.2017.17

[B99] Tayeb Z. Fedjaev J. Ghaboosi N. Richter C. Everding L. Qu X. (2019). Validating deep neural networks for online decoding of motor imagery movements from EEG signals. *Sensors* 19:210. 10.3390/s19010210 30626132 PMC6338892

[B100] Thodoroff P. Pineau J. Lim A. (2016). “Learning robust features using deep learning for automatic seizure detection,” in *Proceedings of the Machine Learning for Healthcare Conference*, Los Angeles, CA.

[B101] Tobimatsu S. Celesia G. G. (2006). Studies of human visual pathophysiology with visual evoked potentials. *Clin Neurophysiol.* 117 1414–1433. 10.1016/j.clinph.2006.01.004 16516551

[B102] Touryan J. Apker G. Lance B. J. Kerick S. E. Ries A. J. McDowell K. (2014). Estimating endogenous changes in task performance from EEG. *Front. Neurosci.* 8:155. 10.3389/fnins.2014.00155 24994968 PMC4061490

[B103] Truong N. D. Kuhlmann L. Bonyadi M. R. Kavehei O. (2018). Semi-supervised seizure prediction with generative adversarial networks. *arXiv* [Preprint] arXiv:1806.08235 10.1109/ACCESS.2019.2944691 31946376

[B104] Tsiouris KM Pezoulas V. C. Zervakis M. Konitsiotis S. Koutsouris D. D. Fotiadis D. I. (2018). A long short-term memory deep learning network for the prediction of epileptic seizures using EEG signals. *Comput. Biol. Med.* 99 24–37. 10.1016/j.compbiomed.2018.05.019 29807250

[B105] Ullah I. Hussain M. Aboalsamh H. (2018). An automated system for epilepsy detection using EEG brain signals based on deep learning approach. *Expert Syst. Appl.* 107 61–71. 10.1016/j.eswa.2018.04.021

[B106] Villena-González M. Palacios-García I. Rodríguez E. López V. (2018). Beta oscillations distinguish between two forms of mental imagery while gamma and theta activity reflects auditory attention. *Front. Hum. Neurosci.* 12:389. 10.3389/fnhum.2018.00389 30337865 PMC6178143

[B107] Wang F. Zhong S. H. Peng J. Jiang J. Yan L. (2018). “Data augmentation for EEG-based emotion recognition with deep convolutional neural networks,” in *MultiMedia Modeling*, eds Schoeffmann K. (Cham: Springer). 10.1007/978-3-319-73600-6_8

[B108] Wei Z. Zou J. Zhang J. Xu J. (2019). Automatic epileptic EEG detection using convolutional neural network with improvements in time-domain. *Biomed. Signal Process. Control* 53:101551. 10.1016/j.bspc.2019.04.028

[B109] Williams J. M. Samal A. Rao P. K. Johnson M. R. (2020). Paired trial classification: a novel deep learning technique for MVPA. *Front. Neuron* 14:417. 10.3389/fnins.2020.00417 32425753 PMC7203477

[B110] Wu Z. Lai Y. Wu D. Yao D. (2008). Stimulator selection in SSVEP-based BCI. *Med. Eng. Phys.* 30 1079–1088. 10.1016/j.medengphy.2008.01.004 18316226

[B111] Xie S. Yang T. Wang X. Monaghan J. (2015). “. Hyper-class augmented and regularized deep learning for fine-grained image classification,” in *Proceedings of the IEEE Conference on Computer Vision & Pattern Recognition*, Boston, MA. 10.1109/CVPR.2015.7298880

[B112] Yang B. Fan C. Guan C. Gu X. Zheng M. (2019). “A framework on optimization strategy for EEG motor imagery recognition,” in *Proceedings of the 2019 41st Annual International Conference of the IEEE Engineering in Medicine and Biology Society (EMBC)*, Berlin. 10.1109/EMBC.2019.8857672 31946010

[B113] Yang D. Liu Y. Zhou Z. Yang Y. Xinbin L. (2020). Decoding visual motions from EEG using attention based RNN. *Appl. Sci.* 10:5662. 10.3390/app10165662

[B114] Yang Q. Yan P. Zhang Y. Yu H. Shi Y. Mou X. (2018). Low-dose CT image denoising using a generative adversarial network with wasserstein distance and perceptual loss. *IEEE Trans. Med. Imaging* 37 1348–1357. 10.1109/TMI.2018.2827462 29870364 PMC6021013

[B115] Yin Z. Zhang J. (2017b). Cross-subject recognition of operator functional states via EEG and switching deep belief networks with adaptive weights. *Neurocomputing* 260 349–366. 10.1016/j.neucom.2017.05.002

[B116] Yin Z. Zhang J. (2017a). Cross-session classification of mental workload levels using EEG and an adaptive deep learning model. *Biomed. Signal Process. Control* 33 30–47. 10.1016/j.bspc.2016.11.013

[B117] Yu K. Xu W. Gong Y. (2008). “Deep learning with kernel regularization for visual recognition,” in *Proceedings of the Conference on Neural Information Processing Systems. DBLP*, Vancouver, BC.

[B118] Yun K. Yu K. Osborne J. Eldin S. Lu T. (2019). Improved visible to IR image transformation using synthetic data augmentation with cycle-consistent adversarial networks. *arXiv* [Preprint] arXiv:1904.11620 [eess.IV] 10.1117/12.2519121

[B119] Zander T. O. Kothe C. Welke S. Roetting M. (2009). “Utilizing secondary input from passive brain-computer interfaces for enhancing human-machine interaction,” in *Proceedings of the International Conference on Foundations of Augmented Cognition Neuroergonomics & Operational Neuroscience*, Berlin: Springer. 10.1007/978-3-642-02812-0_86

[B120] Zhang C. Kim Y. K. Eskandarian A. (2021). EEG-inception: an accurate and robust end-to-end neural network for EEG-based motor imagery classification. *J. Neural Eng.* 18:046014. 10.1088/1741-2552/abed81 33691299

[B121] Zhang D. Yao L. Chen K. Monaghan J. J. M. (2019). A convolutional recurrent attention model for subject-independent EEG signal analysis. *IEEE Signal Process. Lett.* 26 715–719. 10.1109/LSP.2019.2906824

[B122] Zhang D. Yao L. Chen K. Wang S. (2018). “Ready for use: subject-independent movement intention recognition via a convolutional attention model,” in *Proceedings of the 27th ACM International Conference on Information and Knowledge Management (CIKM18). ACM*, Turin. 10.1145/3269206.3269259

[B123] Zhang K. Xu G. Han Z. Ma K. Xiao Z. Chen L. (2020). Data augmentation for motor imagery signal classification based on a hybrid neural network. *Sensors* 20:4485. 10.3390/s20164485 32796607 PMC7474427

[B124] Zhang Q. Liu Y. (2018). Improving brain computer interface performance by data augmentation with conditional deep convolutional generative adversarial networks. *arXiv* [Preprint] arXiv:1806.07108 [cs.HC]

[B125] Zhang X. Wang Z. Liu D. Ling Q. (2018). DADA: deep adversarial data augmentation for extremely low data regime classification. *arXiv* [Preprint] arXiv:1809.00981 10.1109/ICASSP.2019.8683197

[B126] Zhang Z. Duan F. Solé-Casals J. Dinares-Ferran J. Cichocki A. Yang Z. (2019). A novel deep learning approach with data augmentation to classify motor imagery signals. *IEEE Access* 7:15945. 10.1109/ACCESS.2019.2895133

[B127] Zheng W. L. Lu B. L. (2015). Investigating critical frequency bands and channels for EEG-based emotion recognition with deep neural networks. *IEEE Trans. Auton. Ment. Dev.* 7:1. 10.1109/TAMD.2015.2431497

[B128] Zhu X. Liu Y. Li J. Tao W. Qin Z. (2018). “Emotion classification with data augmentation using generative adversarial networks,” in *Advances in Knowledge Discovery and Data Mining*, eds Phung D. Tseng V. Webb G. Ho B. Ganji M. Rashidi L. (Cham: Springer). 10.1007/978-3-319-93040-4_28

